# Dichlorido(2,9-dimethyl-1,10-phenanthroline-κ^2^ *N*,*N*′)mercury(II)

**DOI:** 10.1107/S1600536813001086

**Published:** 2013-01-19

**Authors:** Ismail Warad, Mousa Al-Noaimi, Salim F. Haddad, Rema Othman

**Affiliations:** a Department of Chemistry, AN-Najah National University, Nablus, Palestinian Territories; b Department of Chemistry, Hashemite University, Zarqa 13115, Jordan; c Department of Chemistry, The University of Jordan, Amman 11942, Jordan; d Language Centre, Hashemite University, Zarqa 13115, Jordan

## Abstract

The title compound, [HgCl_2_(C_14_H_12_N_2_)], consists of one 2,9-dimethyl-1,10-phenanthroline (dmphen) ligand chelating the Hg^II^ ion and two chloride ligands coordinating to the Hg^II^ ion, forming a distorted tetra­hedral environment. The dmphen ligand is nearly planar (r.m.s. deviation = 0.0225 Å). The dihedral angle between the normal to the plane defined by the Hg^II^ atom and the two Cl atoms and the normal to the plane of the dmphen ring is 81.8 (1)°.

## Related literature

For related structures, see Alizadeh (2009 ▶); Alizadeh *et al.* (2009 ▶); Wang & Zhong (2009 ▶); Warad *et al.* (2011 ▶). For properties and application of mercury(II) complexes, see: Ramazani *et al.* (2005 ▶); Mahjoub *et al.* (2004 ▶); Canty & Maker (1976 ▶); Canty & Lee (1982 ▶).
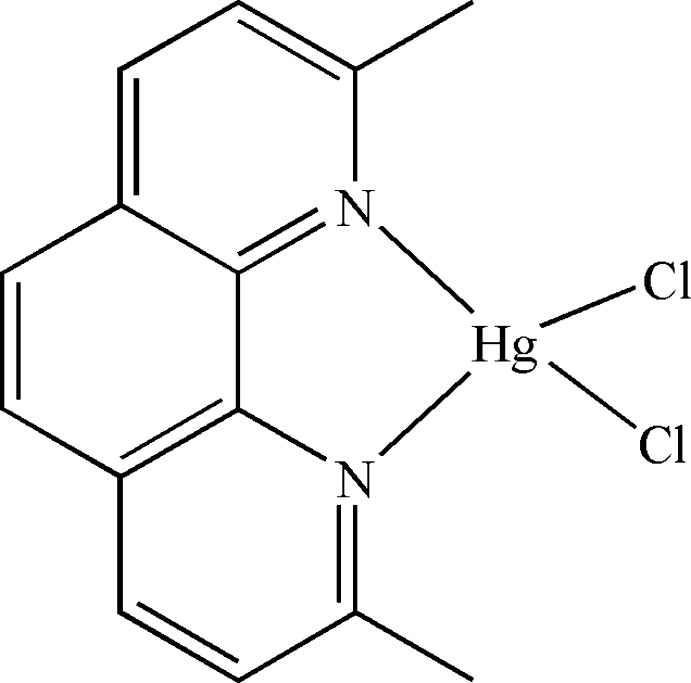


## Experimental

### 

#### Crystal data

[HgCl_2_(C_14_H_12_N_2_)]
*M* *_r_* = 479.75
Monoclinic, 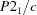

*a* = 7.5732 (13) Å
*b* = 10.3733 (16) Å
*c* = 18.673 (2) Å
β = 94.308 (12)°
*V* = 1462.8 (4) Å^3^
*Z* = 4
Mo *K*α radiation
μ = 10.87 mm^−1^
*T* = 293 K
0.22 × 0.20 × 0.18 mm

#### Data collection

Agilent Xcalibur Eos diffractometer
Absorption correction: multi-scan (*CrysAlis PRO*; Agilent, 2011 ▶) *T*_min_ = 0.106, *T*_max_ = 0.140
5483 measured reflections
2564 independent reflections
1758 reflections with *I* > 2σ(*I*)
*R*_int_ = 0.061

#### Refinement

*R*[*F* ^2^ > 2σ(*F* ^2^)] = 0.051
*wR*(*F* ^2^) = 0.128
*S* = 0.99
2564 reflections
174 parameters
H-atom parameters constrained
Δρ_max_ = 1.81 e Å^−3^
Δρ_min_ = −1.83 e Å^−3^

### 

Data collection: *CrysAlis PRO* (Agilent, 2011 ▶); cell refinement: *CrysAlis PRO*; data reduction: *CrysAlis PRO*; program(s) used to solve structure: *SHELXS97* (Sheldrick, 2008 ▶); program(s) used to refine structure: *SHELXL97* (Sheldrick, 2008 ▶); molecular graphics: *ORTEPIII* (Burnett & Johnson, 1996 ▶); software used to prepare material for publication: *SHELXTL* (Sheldrick, 2008 ▶).

## Supplementary Material

Crystal structure: contains datablock(s) I, global. DOI: 10.1107/S1600536813001086/br2220sup1.cif

Structure factors: contains datablock(s) I. DOI: 10.1107/S1600536813001086/br2220Isup2.hkl

Additional supplementary materials:  crystallographic information; 3D view; checkCIF report

## Figures and Tables

**Fig. 1.**
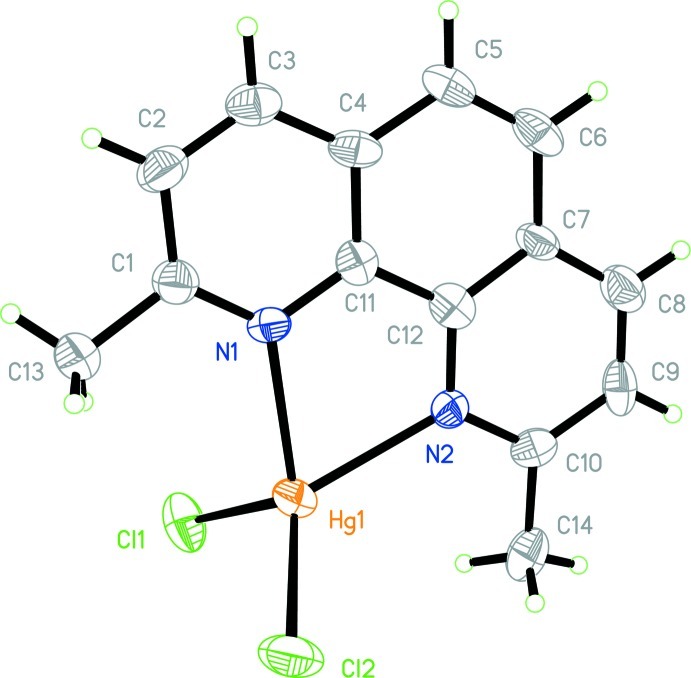
An ORTEP (Burnett & Johnson, 1996) view of Hg(Cl)2(dmphen). Displacement ellipsoids are drawn at the 30% probability level. H atoms are represented as small spheres of arbitrary radii.

## supplementary crystallographic information

### Comment

The coordination chemistry of mercury(II) with N-donor ligands is of interest due to applications as solid-state materials (Ramazani *et al.*, 2005; Mahjoub *et al.*, 2004). Hg(II) complexes with bidentate ligands have been obtained in which Hg(II) adopts higher coordination numbers such as complexes of 1,10-phenanthroline (Canty & Maker, 1976) and N-substituted pyrazole (Canty & Lee, 1982). The molecular structure of [HgCl_2_(C_14_H_12_N_2_)], along with the numbering scheme is shown in Fig. 1. HgCl_2_ is chelated by the bidentate phenanthroline molecule and that the coordination of the nitrogen and chlorine atoms about the Hg atom is essentially a distorted tetrahedral environment (Fig. 1).

### Experimental

The desired complex was prepared by mixting of mercury chloride (HgCl_2_, 39.7 mg,0.14 mmol) in methanol (10 ml) with dmphen (32.0 mg, 0.15 mmol) in dichloromethane (5 ml) is stirred for one houre at room temperature. The obtained solution was concentrated to about 2 ml underreduced pressure and mixed to 30 ml of diethyl ether. The white precipitate was filtered and dried. suitable colourless crystals were obtained by slow diffusion of diethyl ether into a solution of the complex in dichloromethane.

### Refinement

All nonhydrogen atoms were refined anisotropically.H atoms were positioned geometrically, with C-H = 0.93 and 0.96 Å for aromatic and methyl H, respectively, and constrained to ride on their parent atoms, with *U*_iso_(H) = 1.2Ueq(C). Highest difference peak and hole are 1.81 and -1.83e/Å^3 ^ close to the Hg atom.

### Crystal data

**Table:**

[HgCl_2_(C_14_H_12_N_2_)]	*F*(000) = 896
*M**_r_* = 479.75	*D*_x_ = 2.178 Mg m^−^^3^
Monoclinic, *P*2_1_/*c*	Mo *K*α radiation, λ = 0.71073 Å
Hall symbol: -P 2ybc	Cell parameters from 1534 reflections
*a* = 7.5732 (13) Å	θ = 2.9–29.0°
*b* = 10.3733 (16) Å	µ = 10.87 mm^−^^1^
*c* = 18.673 (2) Å	*T* = 293 K
β = 94.308 (12)°	Block, colourless
*V* = 1462.8 (4) Å^3^	0.22 × 0.20 × 0.18 mm
*Z* = 4	

### Data collection

**Table:**

Agilent Xcalibur Eos diffractometer	2564 independent reflections
Radiation source: Enhance (Mo) X-ray Source	1758 reflections with *I* > 2σ(*I*)
Graphite monochromator	*R*_int_ = 0.061
Detector resolution: 16.0534 pixels mm^-1^	θ_max_ = 25.0°, θ_min_ = 2.9°
ω scans	*h* = −9→7
Absorption correction: multi-scan (*CrysAlis PRO*; Agilent, 2011)	*k* = −12→12
*T*_min_ = 0.106, *T*_max_ = 0.140	*l* = −16→22
5483 measured reflections	

### Refinement

**Table:**

Refinement on *F*^2^	Primary atom site location: structure-invariant direct methods
Least-squares matrix: full	Secondary atom site location: difference Fourier map
*R*[*F*^2^ > 2σ(*F*^2^)] = 0.051	Hydrogen site location: inferred from neighbouring sites
*wR*(*F*^2^) = 0.128	H-atom parameters constrained
*S* = 0.99	*w* = 1/[σ^2^(*F*_o_^2^) + (0.0521*P*)^2^] where *P* = (*F*_o_^2^ + 2*F*_c_^2^)/3
2564 reflections	(Δ/σ)_max_ < 0.001
174 parameters	Δρ_max_ = 1.81 e Å^−^^3^
0 restraints	Δρ_min_ = −1.83 e Å^−^^3^

### Special details

**Table:**

Geometry. All e.s.d.'s (except the e.s.d. in the dihedral angle between two l.s. planes) are estimated using the full covariance matrix. The cell e.s.d.'s are taken into account individually in the estimation of e.s.d.'s in distances, angles and torsion angles; correlations between e.s.d.'s in cell parameters are only used when they are defined by crystal symmetry. An approximate (isotropic) treatment of cell e.s.d.'s is used for estimating e.s.d.'s involving l.s. planes.
Refinement. Refinement of *F*^2^ against ALL reflections. The weighted *R*-factor *wR* and goodness of fit *S* are based on *F*^2^, conventional *R*-factors *R* are based on *F*, with *F* set to zero for negative *F*^2^. The threshold expression of *F*^2^ > σ(*F*^2^) is used only for calculating *R*-factors(gt) *etc*. and is not relevant to the choice of reflections for refinement. *R*-factors based on *F*^2^ are statistically about twice as large as those based on *F*, and *R*- factors based on ALL data will be even larger.

### Fractional atomic coordinates and isotropic or equivalent isotropic displacement parameters (Å^2^)

**Table:**

	*x*	*y*	*z*	*U*_iso_*/*U*_eq_	
Hg1	0.21008 (6)	0.28289 (4)	0.39470 (2)	0.0597 (2)	
Cl1	−0.0404 (4)	0.3290 (4)	0.31096 (16)	0.0818 (10)	
Cl2	0.4266 (5)	0.4523 (3)	0.39503 (19)	0.0871 (11)	
C5	0.3385 (16)	−0.2144 (11)	0.4986 (7)	0.063 (3)	
H5A	0.3736	−0.3002	0.4975	0.076*	
N1	0.2952 (11)	0.0704 (7)	0.3787 (4)	0.047 (2)	
C1	0.3465 (14)	0.0196 (11)	0.3185 (6)	0.057 (3)	
C10	0.1290 (15)	0.2260 (10)	0.5625 (7)	0.055 (3)	
C11	0.2939 (12)	−0.0049 (11)	0.4375 (5)	0.048 (2)	
C3	0.3905 (15)	−0.1866 (12)	0.3704 (7)	0.060 (3)	
H3A	0.4210	−0.2731	0.3670	0.072*	
C8	0.1676 (15)	0.0244 (12)	0.6240 (6)	0.067 (3)	
H8A	0.1636	−0.0247	0.6655	0.080*	
C12	0.2350 (11)	0.0470 (11)	0.5026 (5)	0.048 (3)	
N2	0.1849 (11)	0.1747 (8)	0.5019 (4)	0.045 (2)	
C7	0.2276 (13)	−0.0328 (10)	0.5619 (6)	0.052 (3)	
C14	0.0775 (16)	0.3651 (12)	0.5595 (6)	0.073 (4)	
H14A	0.1822	0.4176	0.5615	0.110*	
H14B	0.0100	0.3853	0.5995	0.110*	
H14C	0.0073	0.3819	0.5155	0.110*	
C4	0.3430 (13)	−0.1380 (10)	0.4363 (6)	0.052 (3)	
C2	0.3929 (15)	−0.1104 (11)	0.3118 (7)	0.066 (3)	
H2A	0.4245	−0.1434	0.2683	0.080*	
C6	0.2834 (14)	−0.1637 (11)	0.5598 (7)	0.062 (3)	
H6A	0.2820	−0.2147	0.6007	0.074*	
C9	0.1155 (14)	0.1497 (14)	0.6246 (5)	0.061 (3)	
H9A	0.0715	0.1850	0.6654	0.074*	
C13	0.3475 (18)	0.1076 (12)	0.2547 (6)	0.083 (4)	
H13A	0.3449	0.0572	0.2116	0.124*	
H13B	0.4528	0.1594	0.2587	0.124*	
H13C	0.2453	0.1626	0.2532	0.124*	

### Atomic displacement parameters (Å^2^)

**Table:**

	*U*^11^	*U*^22^	*U*^33^	*U*^12^	*U*^13^	*U*^23^
Hg1	0.0813 (4)	0.0419 (4)	0.0565 (3)	0.0057 (2)	0.0083 (3)	0.00836 (19)
Cl1	0.075 (2)	0.111 (3)	0.0590 (19)	0.016 (2)	0.0022 (15)	0.0250 (18)
Cl2	0.106 (3)	0.0474 (18)	0.107 (3)	−0.0145 (19)	0.003 (2)	0.0152 (18)
C5	0.070 (8)	0.046 (8)	0.075 (9)	−0.002 (6)	0.009 (6)	0.023 (7)
N1	0.056 (5)	0.033 (5)	0.051 (5)	−0.001 (4)	0.002 (4)	−0.003 (4)
C1	0.061 (7)	0.052 (7)	0.059 (7)	0.003 (6)	0.014 (5)	0.003 (6)
C10	0.053 (7)	0.047 (7)	0.065 (8)	0.003 (5)	0.007 (5)	−0.008 (6)
C11	0.037 (6)	0.056 (7)	0.049 (6)	−0.003 (5)	−0.001 (4)	0.003 (6)
C3	0.050 (7)	0.049 (7)	0.080 (9)	0.007 (5)	−0.004 (6)	−0.003 (7)
C8	0.071 (8)	0.066 (8)	0.062 (8)	−0.016 (7)	−0.002 (6)	0.017 (7)
C12	0.020 (5)	0.060 (7)	0.062 (7)	−0.006 (5)	−0.007 (4)	0.008 (6)
N2	0.051 (5)	0.043 (5)	0.041 (5)	−0.006 (4)	0.004 (4)	−0.002 (4)
C7	0.062 (7)	0.038 (6)	0.055 (7)	−0.007 (5)	0.001 (5)	0.015 (5)
C14	0.078 (9)	0.088 (10)	0.055 (7)	0.002 (8)	0.015 (6)	−0.021 (7)
C4	0.042 (6)	0.036 (6)	0.077 (8)	−0.007 (5)	0.000 (5)	0.005 (6)
C2	0.076 (9)	0.057 (8)	0.065 (8)	0.015 (7)	−0.001 (6)	−0.016 (7)
C6	0.048 (7)	0.060 (8)	0.075 (9)	−0.014 (6)	−0.006 (6)	0.030 (7)
C9	0.051 (7)	0.099 (10)	0.035 (6)	−0.009 (7)	0.007 (4)	0.005 (7)
C13	0.133 (13)	0.059 (8)	0.061 (8)	0.001 (8)	0.031 (8)	0.006 (7)

### Geometric parameters (Å, º)

**Table:**

Hg1—N2	2.314 (8)	C3—C4	1.401 (15)
Hg1—N1	2.322 (8)	C3—H3A	0.9300
Hg1—Cl2	2.403 (3)	C8—C9	1.359 (16)
Hg1—Cl1	2.414 (3)	C8—C7	1.408 (15)
C5—C6	1.352 (16)	C8—H8A	0.9300
C5—C4	1.410 (15)	C12—N2	1.378 (13)
C5—H5A	0.9300	C12—C7	1.386 (13)
N1—C1	1.326 (12)	C7—C6	1.424 (15)
N1—C11	1.348 (12)	C14—H14A	0.9600
C1—C2	1.402 (15)	C14—H14B	0.9600
C1—C13	1.501 (15)	C14—H14C	0.9600
C10—N2	1.348 (13)	C2—H2A	0.9300
C10—C9	1.414 (15)	C6—H6A	0.9300
C10—C14	1.494 (15)	C9—H9A	0.9300
C11—C4	1.430 (14)	C13—H13A	0.9600
C11—C12	1.432 (13)	C13—H13B	0.9600
C3—C2	1.350 (16)	C13—H13C	0.9600
			
N2—Hg1—N1	72.1 (3)	C10—N2—C12	118.3 (9)
N2—Hg1—Cl2	116.9 (2)	C10—N2—Hg1	125.9 (7)
N1—Hg1—Cl2	119.9 (2)	C12—N2—Hg1	115.8 (6)
N2—Hg1—Cl1	123.0 (2)	C12—C7—C8	116.1 (10)
N1—Hg1—Cl1	108.5 (2)	C12—C7—C6	121.2 (11)
Cl2—Hg1—Cl1	111.07 (13)	C8—C7—C6	122.6 (10)
C6—C5—C4	120.5 (11)	C10—C14—H14A	109.5
C6—C5—H5A	119.8	C10—C14—H14B	109.5
C4—C5—H5A	119.8	H14A—C14—H14B	109.5
C1—N1—C11	118.8 (9)	C10—C14—H14C	109.5
C1—N1—Hg1	126.0 (7)	H14A—C14—H14C	109.5
C11—N1—Hg1	115.2 (7)	H14B—C14—H14C	109.5
N1—C1—C2	123.3 (10)	C3—C4—C5	123.1 (10)
N1—C1—C13	116.8 (10)	C3—C4—C11	116.5 (10)
C2—C1—C13	119.9 (10)	C5—C4—C11	120.4 (10)
N2—C10—C9	120.9 (10)	C3—C2—C1	118.2 (11)
N2—C10—C14	116.5 (10)	C3—C2—H2A	120.9
C9—C10—C14	122.5 (10)	C1—C2—H2A	120.9
N1—C11—C4	121.9 (9)	C5—C6—C7	120.3 (11)
N1—C11—C12	119.7 (10)	C5—C6—H6A	119.8
C4—C11—C12	118.4 (10)	C7—C6—H6A	119.8
C2—C3—C4	121.4 (11)	C8—C9—C10	119.3 (11)
C2—C3—H3A	119.3	C8—C9—H9A	120.3
C4—C3—H3A	119.3	C10—C9—H9A	120.3
C9—C8—C7	121.5 (11)	C1—C13—H13A	109.5
C9—C8—H8A	119.3	C1—C13—H13B	109.5
C7—C8—H8A	119.3	H13A—C13—H13B	109.5
N2—C12—C7	123.7 (10)	C1—C13—H13C	109.5
N2—C12—C11	117.1 (9)	H13A—C13—H13C	109.5
C7—C12—C11	119.1 (10)	H13B—C13—H13C	109.5
